# A comparison study of percutaneous endoscopic decompression and posterior decompressive laminectomy in the treatment of thoracic spinal stenosis

**DOI:** 10.1186/s12891-020-03739-8

**Published:** 2020-11-03

**Authors:** Xiao-Kang Cheng, Fu-Cheng Bian, Zhao-Yu Liu, Feng-Kai Yang, Bin Chen

**Affiliations:** 1 Department of Spine Surgery, Chengde Medical University Affiliated Hospital, Chengde, 067000 Hebei Province China; 2 Chengde Medical University, Chengde, 067000 Hebei China

**Keywords:** Thoracic spinal stenosis (TSS), Percutaneous endoscopic decompression (PED), Posterior decompressive laminectomy (PDL), Thoracic myelopathy

## Abstract

**Background:**

Percutaneous endoscopic decompression (PED) is considered a minimally invasive and safe procedure in lumbar degenerative disease. Few authors report the success of PED for thoracic spinal stenosis (TSS) with thoracic myelopathy. The objective of this study was to compare the outcome of PED versus posterior decompressive laminectomy (PDL) for TSS.

**Methods:**

We retrospectively reviewed 30 consecutive patients who underwent surgery for single-level TSS from January 1, 2015 to May 1, 2019.These patients were divided into PED (*n* = 16) and PDL(*n* = 14) group. Preoperative demographic characteristics and perioperative outcomes were reviewed. Pre- and postoperative neurological status was evaluated using the modified Japanese Orthopaedic Association (mJOA) score and the recovery rate (RR).

**Results:**

The patients’ mean age was 57.3 years (27–76) in PED group and 58.8 years (34–77) in PDL group. No statistical difference was found between two groups with regards to neurological status at pre-operative and final follow-up. The RR in PED group achieved the same improvement as PDL group (87.5% vs 85.7%, *P* > 0.05), while the PED brought advantages in operative time(m) (86.4 vs 132.1, *p* < 0.05), blood loss (mL) (18.21 vs 228.57, *p* < 0.05),drainage volume(mL) (15.5 vs 601.4, *p* < 0.05), and hospital stay (d) (3.6 vs 5.6, *p* < 0.05).

**Conclusions:**

Both PED and PDL showed favorable outcome in the treatment of TSS. Besides, PED had advantages in reducing traumatization. In terms of perioperative quality of life, PED could be an efficient supplement to traditional posterior decompressive laminectomy in patients with TSS.

## Background

Thoracic spinal stenosis (TSS) with thoracic myelopathy. Usually caused by thoracic ossification of the posterior longitudinal ligament or ligamentum flavum, and intervertebral disc herniation, is a reduction of the spinal canal with associated compression of the thoracic spinal cord [1]. Diagnosis is often difficult owing to myriad presenting symptoms including difficulty walking, local pain, sphincter dysfunctions, and lower extremity numbness and weakness [2].

The major studies show that conservative therapy is not effective in TSS with myelopathy to prevent the sequelae of spinal cord compression. Traditional open spinal decompression, involving spinous process and paraspinal muscle dissection, en bloc resection of lamina and removal of the intervertebral disc herniation and hyperplastic ligament, is the prevailing therapy [3, 4]. Although with positive results, there are risks of kyphosis, the adjacent segment degeneration (ASD),and catastrophic neurological deterioration [5]. Based on improvements in optical technology and equipment, good outcomes and advantages of PED for lumbar degenerative disease have been used in recent years [6, 7] . However, there were few reports of PED for the treatment of TSS [8, 9]. The objective of this study was to compare the outcome of PED versus PDL for TSS.

## Methods

### Patient population

This study was approved by the Ethics Committee of our hospital, written informed consent was obtained. From January 1, 2015 and May 1, 2019, 30 consecutive patients underwent surgery for single-level TSS, including 16 patients who underwent PED and 14 patients who underwent PDL. Inclusion criteria: patients with single-segment TSS confirmed by MRI and CT. Exclusion criteria: Combined with lumbar or cervical degenerative disease. Preoperative demographic characteristics, perioperative outcomes and neurological status were recorded. The full percutaneous endoscopic spine system, bipolar radiofrequency system, and endoscopic instruments (MaxMorespine GmbH, Germany) were used in PED.

### Surgical technique

**Fig. 1:**
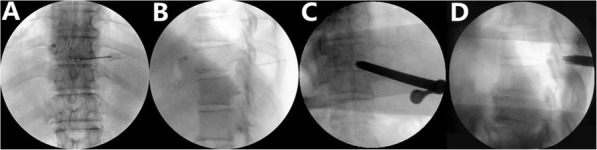
The Spinal needle and bevelled cannula. **a c** The needle and cannula was placed on medial margin in anteroposterior view. **b d** The needle and cannula was placed on the lamina in lateral view

**Fig. 2:**
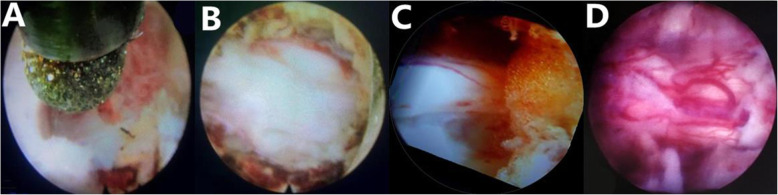
Intraoperative views. **a** The diamond abrasor drilling the lamina. **b** After the OLF were removed by endoscopic instruments, the dura sac was visible. **c** The nerve root was exposed. **d** The whole dura sac was exposed (white area) and fluctuated well

For PED group, the surgical procedure was performed below (Based on T10–11 Segment of OLF):

After positioning in the prone position, local anesthesia was used to allow for communication between the surgeon and patient during the procedure. The entry point was 3–5 cm from the midline. After anesthesia the skin, a spinal needle was introduced (Fig. 1). The needle was positioned at the trailing edge of the lamina and at the medial margin of the facet joint. A series of obturators were inserted along the needle. A bevelled cannula was inserted along the obturators. The procedure was conducted with solution. After removing the tissue attached with the lamina with the endoscopic instruments, an endoscopic drill was used to expose the lamina completely. After that, a diamond drill was used to thin the OLF (Fig. 2). Then the endo forceps were used to remove the OLF carefully untill the bilateral sides of dural sac and nerve roots were checked and exposed. All endoscopic instruments were taken out, and the incision was closed with or without a suction drainage.

For PDL group, the surgical procedure was performed as described below (en bloc decompression):

The surgical method and steps were consistent with that reported in the literature [4]. To reduce the edema, inflammation and avoid neurological dysfunction, anti-inflammatory medication and neuronutrition were given after operation.

### Evaluation indicators

Preoperative demographic characteristics and perioperative outcomes were compared. Neurological status were evaluated using the mJOA score. Neurological recovery was assessed based on the RR. The RR = (postoperative - preoperative mJOA score)/(11-preoperative mJOA score) × 100. A score of 0–24% was poor; 25–49%, fair; 50–74%, good; and 75–100%, excellent [10].

## Statistical method

Statistical assessments were analyzed using the SPSS 21 program (USA, IBM corporation). The independent samples t-test and chi-squared test were used to evaluate preoperative demographic characteristics and perioperative outcomes, and repeated-measures analysis of variance was used to evaluate mJOA between the two groups. *P* < 0.05 was considered significant.

## Results

### Perioperative outcomes and preoperative demographic characteristics

We kept in contact with all of the 30 patients (16 in PED group and 14 in PDL group) throughout the follow-up. Preoperative demographic characteristics were similar in the two groups as shown in Table 1. Perioperative outcomes, including operative time, blood loss, drainage volume and hospital stay time, were recorded as shown in Table 2. PED had advantages in operative time (minutes) (86.4 vs 132.1, *p* < 0.05), blood loss (mL) (18.2 vs 228.6, *p* < 0.05),drainage volume (mL) (15.5 vs 601.4, *p* < 0.05), hospital stay (days) (3.6 vs 5.6, *p* < 0.05).

**Table 1:** Preoperative demographic characteristics

Characteristics	PED Group(*n* = 16)	PDL Group (*n* = 14)	*P* Value
Age (years)	57.3 ± 14.4	58.8 ± 9.4	> 0.05
Sex (male)	9	6	> 0.05
Duration of symptoms (months)	7.1 ± 7.4	6.1 ± 7.9	> 0.05
Level			> 0.05
T6–7	0	2	
T8–9	2	1	
T9–10	2	0	
T10–11	3	4	
T11–12	9	7	
Comorbidities	13/16	13/14	> 0.05
Hypertension	8	8	
Diabetes	3	3	
Heart diseases	1	2	
Pulmonary	2	1

**Table 2:** Perioperative outcomes

Characteristics	PED Group(*n* = 16)	PDL Group (*n* = 14)	*P* Value
Blood loss (mL)	18.2 ± 3.2	228.6 ± 120.4	0.00
Duration of surgery (minutes)	86.4 ± 18.2	132.1 ± 19.7	0.00
Hospital stay (day)	3.6 ± 0.8	5.6 ± 1.7	0.00
Drainage (mL)	15.5 ± 6.9	601.4 ± 371.7	0.00
Complications	1/16	3/14	> 0.05

### Clinical results

**Fig. 3:**
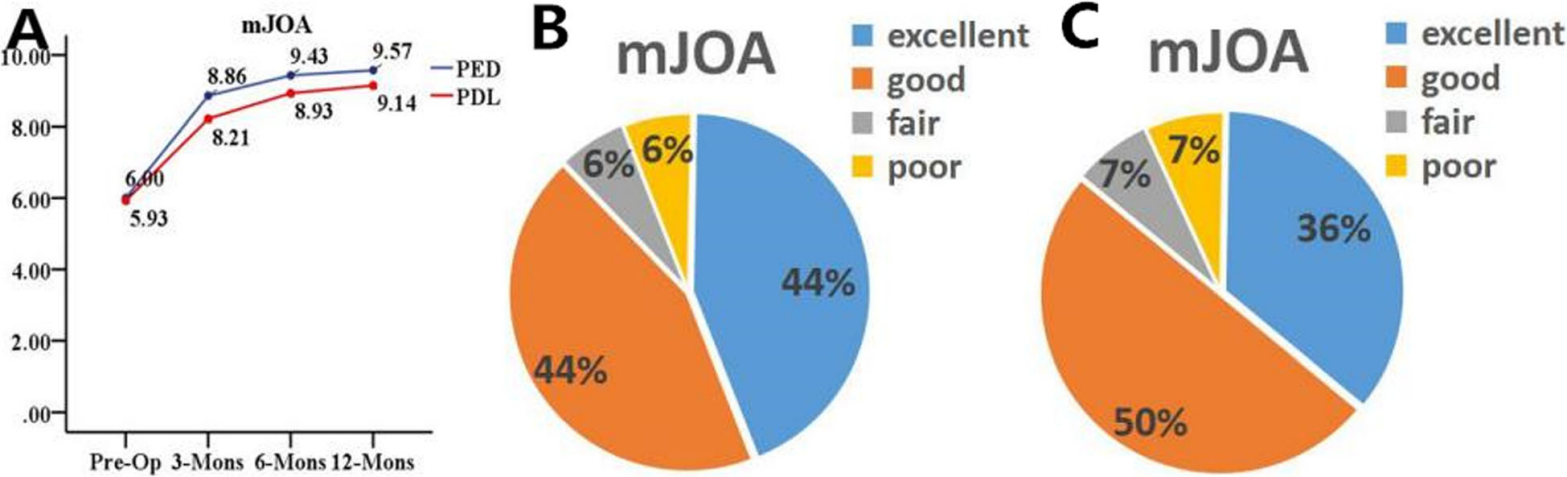
Preoperative views of CT and MRI revealed OLF

Statistically improvements in clinical outcomes before surgery to 3, 6, and 12 months after surgery were estimated by the mJOA score and RR (Fig. 3). Both groups had similar improvement in the mean mJOA scores at each follow-up time point (*P* < 0.05). In the preoperative stage, the mean mJOA scores were similar between the 2 groups (6.0 ± 1.4 vs5.9 ± 0.9, *P* < 0.05). The mean mJOA scores of the PED group improved to 9.5 ± 1.3 at the last follow-up (*P* < 0.05). The mJOA scores also improved significantly in the PDL group to 9.1 ± 1.0 at the last follow-up (*P* < 0.05). Moreover, there were no intergroup differences at any follow-up time point between groups (*P* > 0.05). Good-to-excellent rate was no differences at the final follow-up(87.5% vs 85.7%, *P* > 0.05).

### Complications

In the PED group, one case had dural tear but no cerebrospinal fluid leakage occurred. In PDL group, one patient had dural tear and cerebrospinal fluid leakage. One patient had cerebrospinal fluid leakage and incision infection, which resolved after debridement and antibiotic therapy. One case experienced neurological deterioration. There were no reports of cyst or incision dehiscence, thrombophlebitis,kyphosis or vascular injury during their follow-up.

## Discussion

The aim of this retrospective research was to evaluate and compare the outcome of PED and PDL in geriatric patients with TSS. Although no significant difference was found in clinical results between both groups, the PED techniques brought advantages in terms of faster recovery, less trauma, and less complications from our own experience.

It has been reported that the lower thoracic spine is the most frequently affected segment while the upper and mid-thoracic vertebrae are rarely affected within the thoracic spine. This propensity was also seen in this retrospective research.

The traditional surgical methods for TSS are laminotomy with or without fusion [4, 11]. The procedures depends on general physical condition of patient and TSS type according to CT and MRI evaluation [12]. Some have recommended laminoplasty for the treatment of TSS. However, laminoplasty, merely expands the volume of the spinal canal and does not remove the posterior longitudinal ligament or ligamentum flavum, and intervertebral disc herniation, is not suitable for severe TSS because the procedure sometimes leads to an insufficient decompression and is technically difficult due to the adherence of the OLF to the dura mater. Furthermore, lamina treated with laminoplasty may return to the preoperative position.

At present, laminectomy with or without fusion is the most popular procedure for TSS [13]. It has been considered an effective method for TSS with myelopathy to undergo PDL with or without fusion [14]. Some authors [15, 16] have recommended laminectomy with fusion in the treatment of TSS for that instability of the thoracic spine was caused by excessive removal of the lamina and facet joint [11], and therefore it sometimes required pedicle screw fixation and fusion. Although laminectomy achieves a good spinal cord decompression, it comes at the price of multiple complications such as increased incidence of acute neurological deterioration, dura tear, and kyphosis. Since the patients are particularly geriatric who frequently have comorbidities, to consider the necessity for more extensive surgery associated with fusion is vital. Besides, some authours have concluded that decompression alone has lower costs than fusion.

Microendoscopic decompression (MED) treating TSS also has been reported by Baba [17]. Although with a surgeon-friendly view, it requires partial facet joint resection and general anesthesia, which is similar to the traditional open approach. So an effective and less invasive surgical technique is warranted. PED has been popular in the treatment of lumbar degenerative disease because of development of endoscopic instruments and increased patient demand [18]. PED is less invasive than MED for selected patients with lumbar degenerative disease [19, 20]. Jia [8] performed the PED for the treatment of one OLF case at T2/3. Miao [9] also reported successful PED in treating two cases with unilateral OLF at T9/10 and T3/4 using the PED with paramedian approach.

**Fig. 4:**
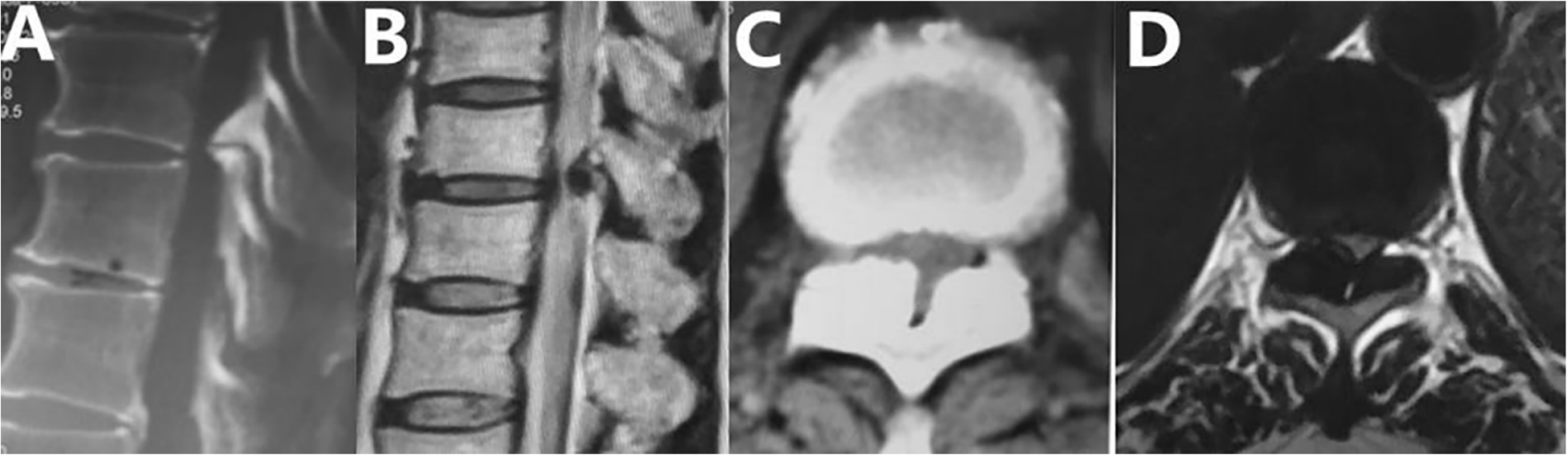
Postoperative CT and MRI. Satisfactory decompression was performed

**Fig. 5:**
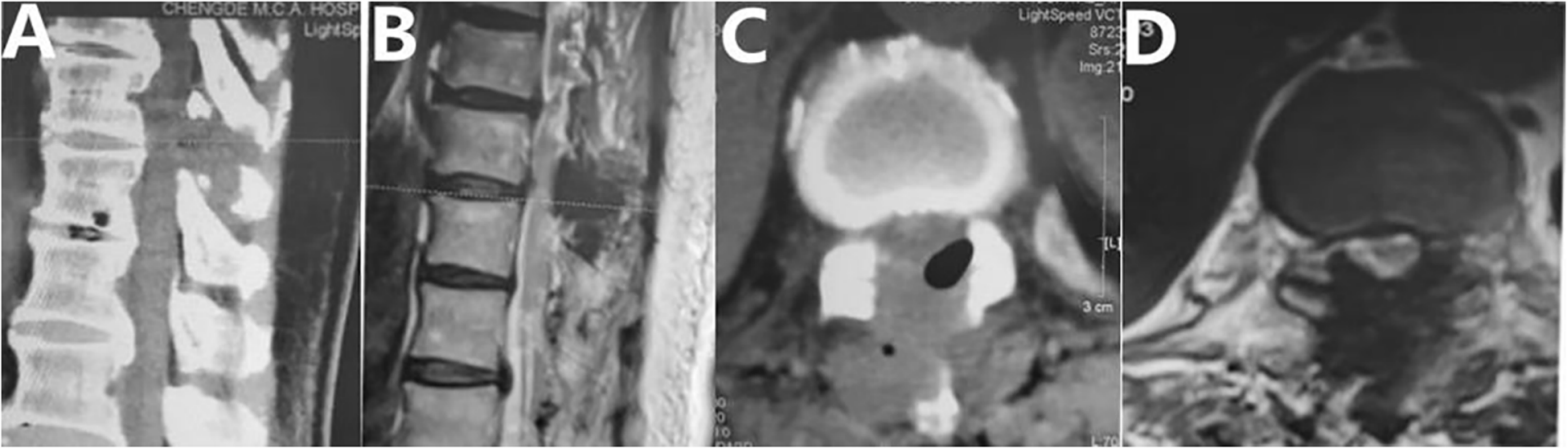
Clinical outcomes. **a** mJOA scores. **b c** Outcome of the RR

The postoperative outcomes showed that preoperative symptoms were relieved and the decompression was completed without severe complications (Figs. 4 and 5). The PED requires only a incision of approximately 7.5 mm, with the advantages of causing little damage to the paraspinal muscles, dissecting less tissue, and reducing operative time and blood loss. Little lamina and facet joint was removed, therefore no instability of the spine was caused. Pedicle screw fixation and fusion may be unnecessary, the medical costs may be reduced. Moreover, PED can reduce the length of hospitalization [21]. Besides, it can provide clear visualization with saline solution, which is helpful in improving safety of the elderly patients [22]. The surgeons can receive feedback during the operation to reduce the risks of anesthesia related complications and avoiding acute neurological deterioration when removing the OLF and disc herniation with endoscopic instruments. For developing countries, it is very benefcial to economically disadvantaged patients if general anaesthesia with neuromonitoring is not necessary. Moreover, compared with PDL, PED can shorten patients recovery time markedly. Therefore, this procedure could be an effective choice for geriatric patients for whom general anesthesia would be harmful. As for patients with OLF, we can use the diamond high-speed drill to thin the OLF with clear and enlarged visualization as called floating method. Some authors suggested that it could be used to avoid dural tears if the floating method were used [23].

Neurological deterioration is a serious complication for TSS [24]. Some authors reported that the incidence of perioperative neurological deterioration was as high as 14.5%. The insertion of even a 1-mm Kerrison rongeur into the thoracic canal may cause catastrophic neurological deterioration. Therefore, it is dangerous to remove the lamina with a Kerrison rongeur in the thoracic spine. So, in the process of thoracic laminectomy and spinal cord decompression, we should be very careful, which may cause more time and bleeding than open lumbar decompression. In PDL group, one case experienced neurological deterioration after recovery from anesthesia. Although with use of methylprednisolone and functional exercises, the patient condition was not satisfied at final follow-up.

One of the most common complications of the traditional open spinal decompression is CSF leakage [24]. Although not repaired, no cerebrospinal fluid leakage occurred after endoscopic decompression for one case who had intraoperative dural tears in the PED group. In the PDL group, two cases had CSF leakage after the operation, one of whom had incision infection, which resolved after debridement and antibiotic therapy. Although the recovery was good, the process was painful.

We do not recommend that beginners perform PED independently for the flat learning curve. However, with proper diagnosis, precise indication, and good training, experienced skilled surgeons can use the PED to treat TSS. The primary limitations of the retrospective study are the relatively small number of patients and following time.

## Conclusion

Both PED and PDL showed favorable outcomes for the treatment of single-level TSS. In terms of perioperative quality of life, PED under local anesthesia can be considered as the effective alternatives to traditional open spinal decompression. However, prospective, randomized, controlled trials with a larger sample size and longer follow-up are required in the future.

## Data Availability

The datasets used and/or analysed during the current study are available from the corresponding author on reasonable request.
